# D-2-hydroxyglutarate is essential for maintaining oncogenic property of mutant IDH-containing cancer cells but dispensable for cell growth

**DOI:** 10.18632/oncotarget.3330

**Published:** 2015-03-25

**Authors:** Shenghong Ma, Bowen Jiang, Wanglong Deng, Zhong-Kai Gu, Fei-Zhen Wu, Tingting Li, Yukun Xia, Hui Yang, Dan Ye, Yue Xiong, Kun-Liang Guan

**Affiliations:** ^1^ State Key Laboratory of Genetic Engineering, Collaborative Innovation Center for Genetics and Development, School of Life Sciences, Molecular and Cell Biology Lab, Institutes of Biomedical Sciences, Shanghai Medical College, Fudan University, Shanghai 200032, China; ^2^ State Key Laboratory of Medical Genomics and Shanghai Institute of Hematology, SJTU-SM, Shanghai 200025, China; ^3^ Lineberger Comprehensive Cancer Center, Department of Biochemistry and Biophysics, University of North Carolina at Chapel Hill, North Carolina 27599, USA; ^4^ Department of Pharmacology and Moores Cancer Center, University of California San Diego, La Jolla, California 92093, USA

**Keywords:** D-2-HG, IDH mutation, D2HGDH, tumorigenesis

## Abstract

Cancer-associated isocitrate dehydrogenase (IDH) 1 and 2 mutations gain a new activity of reducing α-KG to produce D-2-hydroxyglutarate (D-2-HG), which is proposed to function as an oncometabolite by inhibiting α-KG dependent dioxygenases. We investigated the function of D-2-HG in tumorigenesis using IDH1 and IDH2 mutant cancer cell lines. Inhibition of D-2-HG production either by specific deletion of the mutant IDH1-R132C allele or overexpression of D-2-hydroxyglutarate dehydrogenase (D2HGDH) increases α-KG and related metabolites, restores the activity of some α-KG-dependent dioxygenases, and selectively alters gene expression. Ablation of D-2-HG production has no significant effect on cell proliferation and migration, but strongly inhibits anchorage independent growth *in vitro* and tumor growth in xenografted mouse models. Our study identifies a new activity of oncometabolite D-2-HG in promoting tumorigenesis.

## INTRODUCTION

Reprogramming of cellular metabolism has long been hypothesized as one of the key processes during tumorigenesis. It was noted by Otto Warburg that anomalous energy metabolism of high aerobic glycolysis was favored by cancer cells [1]. The underlying molecular mechanism for altered metabolic regulation in cancer cells is much more complicated than expected. To data, there is still some debate as to how metabolism is altered in tumor cells and whether the metabolic switch contributes to, or is merely a consequence of, tumorigenesis. Recent discoveries in the mutations of isocitrate dehydrogenases 1/2 (IDH1/2) in a large number of human cancers have provided a direct link between altered metabolism and cellular transformation and tumorigenesis [2–6]. The mutant IDH1/2 found in human cancer not only have lost their normal catalytic activity but also conferred a neomorphic enzymatic gain-of-function: the NADPH-dependent reduction of α-KG to D-2-hydroxyglutarate (D-2-HG), a trace metabolite in normal cells [7, 8]. Astonishingly, D-2-HG accumulates to as high as 5 – 35 μmol/g (or 5 – 35 mM) in cases of glioma harboring IDH1/2 mutations [7–10]. Taking advantage of its abnormally high accumulation, detecting D-2-HG by magnetic resonance spectroscopy has become an effective way to detect solid tumors harboring IDH1/2 mutations [11, 12].

The cancer-associated mutant IDH1 or IDH2 exclusively produces D-2-HG, but not L-2-HG [7, 8], both of which are byproducts of normal mitochondrial metabolism [13]. To protect cells from high accumulation of 2-HG, there are two FAD-dependent mitochondrial enzymes, D-2-HG dehydrogenase (D2HGDH) and L-2-HG dehydrogenase (L2HGDH), which catalyzes the conversion of harmful D-2-HG and L-2-HG to α-KG [14–16], respectively. Loss-of-function mutations in *D2HGDH* or *L2HGDH* in human cause organic aciduria, as characterized by the high accumulation of D-2-HG or L-2-HG in the urine, respectively [17]. These observations reaffirm the importance of keeping a low level of 2-HG.

Numerous studies have been conducted aiming to understand the function of IDH mutations in cancer, and several hypotheses have been proposed. It has been suggested that IDH mutations change the redox state of cells [18], given that mutant IDH1/2 use NAPDH as a co-factor to catalyze the conversion of α-KG to D-2-HG. More importantly, emerging evidence suggests that IDH mutation derived D-2-HG acts as an oncometabolite to promote cellular transformation, at least in part by inhibiting members of the α-KG-dependent dioxygenase family. We have previously reported that 2-HG functions as an inhibitor towards α-KG-dependent dioxygenases, because D-2-HG is structurally similar to α-KG and can bind to the α-KG binding pocket in these enzymes [19]. In agreement, *in vitro* studies have revealed that D-2-HG inhibits the activity of multiple α-KG-dependent enzymes with a wide range of potencies [19, 20]. Among these α-KG-dependent dioxygenases, the JmjC domain-containing histone demethylases (KDMs) and the TET (ten-eleven translocation) family of DNA hydroxylases have emerged as the two major targets of D-2-HG produced by mutant IDH in promoting tumorigenesis [21].

D-2-HG was reported to promote cytokine-independent growth and block erythropoietin (EPO)-induced differentiation, two properties obligatory for leukemogenesis, in a cell culture model [22]. Notably, depletion of *TET2* also induces growth factor independence and blocks cellular differentiation in TF-1 cells [22]. However, the leukemic transformation is potentiated by cell-permeable D-2-HG, but not L-2-HG. It is unclear why L-2-HG, which is a more potent inhibitor of TET2 and many other α-KG-dependent enzymes than D-2-HG, is ineffective in promoting oncogenic transformation. It has also been reported that mutant IDH or either cell permeable D-2-HG or L-2-HG treatment could lead to the suppression of HNF-4α (a master regulator of hepatocyte identity and quiescence), which is associated with a reduction in histone H3 lysine4 trimethylation (H3K4me3) in its promoter, and block hepatocyte differentiation from progenitors [23]. These data suggest that the oncogenic targets of mutant IDH1/2 might be tumor type specific.

Although the overwhelming genetic evidence of IDH mutation in human cancer unequivocally supports a role of D-2-HG in tumorigenesis, some key questions, such as whether D-2-HG is required only for initiation and/or maintenance of tumorigenic potential, have not been satisfactorily answered. This is because much of previous studies were done using either pharmacological approaches of adding cell permeable D-2-HG or IDH inhibitors or ectopic expression of mutant IDH in already established cancer lines. In this study, we use genetic approach to interrogate the function of D-2-HG using tumor cell lines that naturally harboring the mutant IDH genes. Our results show that D-2-HG levels do not significantly affect cell growth or proliferation, but are critically important in maintaining the tumorigenic property of the mutant IDH-containing cancer cells.

## RESULTS

### D2HGDH overexpression reduces D-2-HG level in *IDH*-mutated cancer cells

Previous studies using cell permeable D-2-HG analogs or mutant IDH inhibitors have the limitation of possible nonspecific effects associated with all pharmacological agents. Moreover, ectopic expression of mutant IDH in established cell lines or even cancer cells may not be pathologically relevant because these cells were transformed through a pathway that is independent of IDH mutation. Therefore, expression of mutant IDH in these cells does not necessarily recapitulate the oncogenesis process caused by IDH mutations. To determine the function of D-2-HG in cancer cells, we aimed to eliminate D-2-HG in human cancer cells that contain a mutant IDH1 or IDH2 allele. Searching of the Cancer Cell Line Encyclopedia (CCLE) database identified two human sarcoma cell lines, HT1080 and SW1353, which contain IDH1^R132C^ and IDH2^R172S^, respectively. By sequencing of genomic DNA, we confirmed the missense mutations of IDH1 Arg-132-Cys (R132C) and IDH2 Arg-172-Ser (R172S) in HT1080 and SW1353 cells, respectively (Figure S1). Consistently, gas chromatography and mass spectrometry (GC-MS) analysis demonstrated that 2-HG was accumulated to high levels in both HT1080 and SW1353 cells (Figures S2A and S2C).

**Figure 1:**
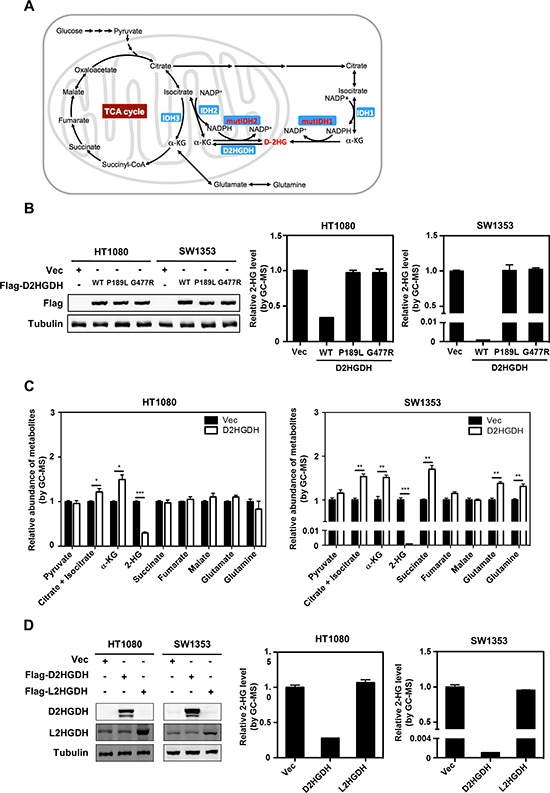
Ectopic expression of D2HGDH reduces D-2-HG in IDH-mutated cancer cells **(A)** A scheme of metabolic pathways involved in D-2-HG metabolism. **(B)** D2HGDH overexpression reduces D-2-HG production in IDH mutant cells. Flag-tagged wild-type or mutant D2HGDH was stably overexpressed in HT1080 and SW1353 cells as indicated. The ectopically expressed proteins were determined by western blot analysis (left panel). Tubulin was used as a loading control. The intracellular concentration of 2-HG was determined by GC-MS analysis as described in “Method” (middle and right panels). **(C)** D2HGDH overexpression increases α-KG levels in IDH mutant cells. Intracellular concentrations of α-KG and other metabolites were determined by GC-MS analysis. **(D)** The effect of 2HGDH on reducing 2-HG is chiral-specific. Flag-tagged D2HGDH or L2HGDH was stably overexpressed in HT1080 and SW1353 cells. The ectopically expressed protein levels were determined by western blot analysis. The L2HGDH antibody likely recognizes the endogenous protein. Shown are average values with standard error (SEM) of triplicated experiments. *denotes *p* < 0.05, **denotes *p* < 0.01, and ***denotes *p* < 0.001 for the indicated comparison.

To determine the effect of D-2-HG, we removed D-2HG in the IDH1 mutant HT1080 cells by two different approaches, overexpression of D2HGDH and deletion of the mutant IDH1^R132C^ allele. We infected the cells with retrovirus expressing Flag-tagged human D2HGDH. We found that stable overexpression of wild-type D2HGDH significantly (*p* < 0.001) reduced 2-HG levels by 67% in HT1080 cells (Figures S2B and 1B). We also examined two D2HGDH mutants, P189L and G477R, found in aciduria patients. Expression of either mutant to a level similar as the wild type D2HGDH failed to reduce 2-HG levels in HT1080 cells (Figure 1B), demonstrating that the patient-associated D2HGDH mutants are catalytically inactive and the D2HGDH enzyme activity is necessary and sufficient to reduce D-2-HG in HT1080. Moreover, stable overexpression of wild-type D2HGDH, but not the P189L or G477R mutant, greatly decreased 2-HG levels by 99.9% in SW1353 cells (Figures 1B and S2D). Although the precise mechanism why D-2HG was much more efficiently reduced in SW1353 than in HT1080 is unclear, the different subcellular localization of IDH1 and IDH2 might contribute to this difference. The mutant IDH1 in HT1080 is in cytoplasm while the mutant IDH2 in SW1353 is in mitochondria. Therefore, the mitochondrially produced D-2-HG may be more efficiently metabolized by the mitochondrially localized D2HGDH in SW1353 cells

In addition to D-2-HG reduction, we found that stable overexpression of D2HGDH also led to a significant increment of α-KG levels in both HT1080 cells (increased by 1.49-fold; *p* < 0.05) and SW1353 cells (increased by 1.53-fold; *p* < 0.01) (Figure 1C), confirming the function of D2HGDH in converting D-2-HG to α-KG. It is known that α-KG acts not only as a key intermediate in the TCA cycle for energy metabolism but also as an entry point for amino acids to enter the TCA cycle (anaplerosis) (Figure 1A). Supporting this notion, we found that in parallel with increased α-KG levels, intracellular levels of citrate and isocitrate, which are the upstream metabolites of α-KG in the TCA cycle, were significantly increased in both HT1080 and SW1353 cells stably overexpressing D2HGDH (increased by 1.22-fold; *p* < 0.05 and 1.51-fold; *p* < 0.01, respectively) (Figure 1C). Additionally, intracellular levels of downstream metabolites of α-KG in the TCA cycle and its anaplerosis were significantly increased in SW1353 cells stably overexpressing D2HGDH, including succinate (increased by 1.70-fold; *p* < 0.01), glutamate (increased by 1.38-fold; *p* < 0.01), and glutamine (increased by 1.30-fold; *p* < 0.01) (Figure 1C). Such increases were, however, not observed in HT1080 cells stably overexpressing D2HGDH (Figure 1C). These findings thus suggest that ectopic expression of D2HGDH in *IDH1/2*-mutated cells can reduce D-2-HG level through facilitating the conversion of D-2-HG to α-KG.

To examine the specificity of 2HGDH, we expressed the human L2HGDH in both HT1080 and SW1353 cells. Stable overexpression of L2HGDH failed to reduce 2-HG level in both cell types in which the endogenous mutant IDH1 exclusively produces D-2-HG (Figure 1D), demonstrating that the effect of D/L-2HGDH on reducing 2-HG is chiral-specific.

### D2HGDH overexpression restores the activity of several α-KG-dependent dioxygenases

We and others have previously shown that D-2-HG acts as an α-KG antagonist to inhibit the activity of α-KG-dependent dioxygenases [19, 20, 24]. We next set out to investigate whether D-2-HG clearance by D2HGDH overexpression could restore the activity of α-KG-dependent dioxygenases in *IDH*-mutated cells. We examined the enzyme activity of PHDs and C-P4Hs in HT1080 and SW1353 cells by measuring their physiological substrates. Our data showed that D2HGDH overexpression in HT1080 and SW1353 cells did not change the protein level of HIF-1α, the substrate of PHDs [25] (Figures S3A and S3B). The PHD inhibitors, such as CoCl_2_, DFO, or DMOG, were included as positive controls. Moreover, we found that overexpression of D2HGDH in HT1080 and SW1353 cells did not change the protein level of Endostatin, the product of C-P4H enzymes (Figure S4). These results suggest that D-2-HG reduction by D2HGDH overexpression in IDH1/2-mutated cells had no significant effect on the activity of PHD and C-P4H enzymes.

**Figure 2:**
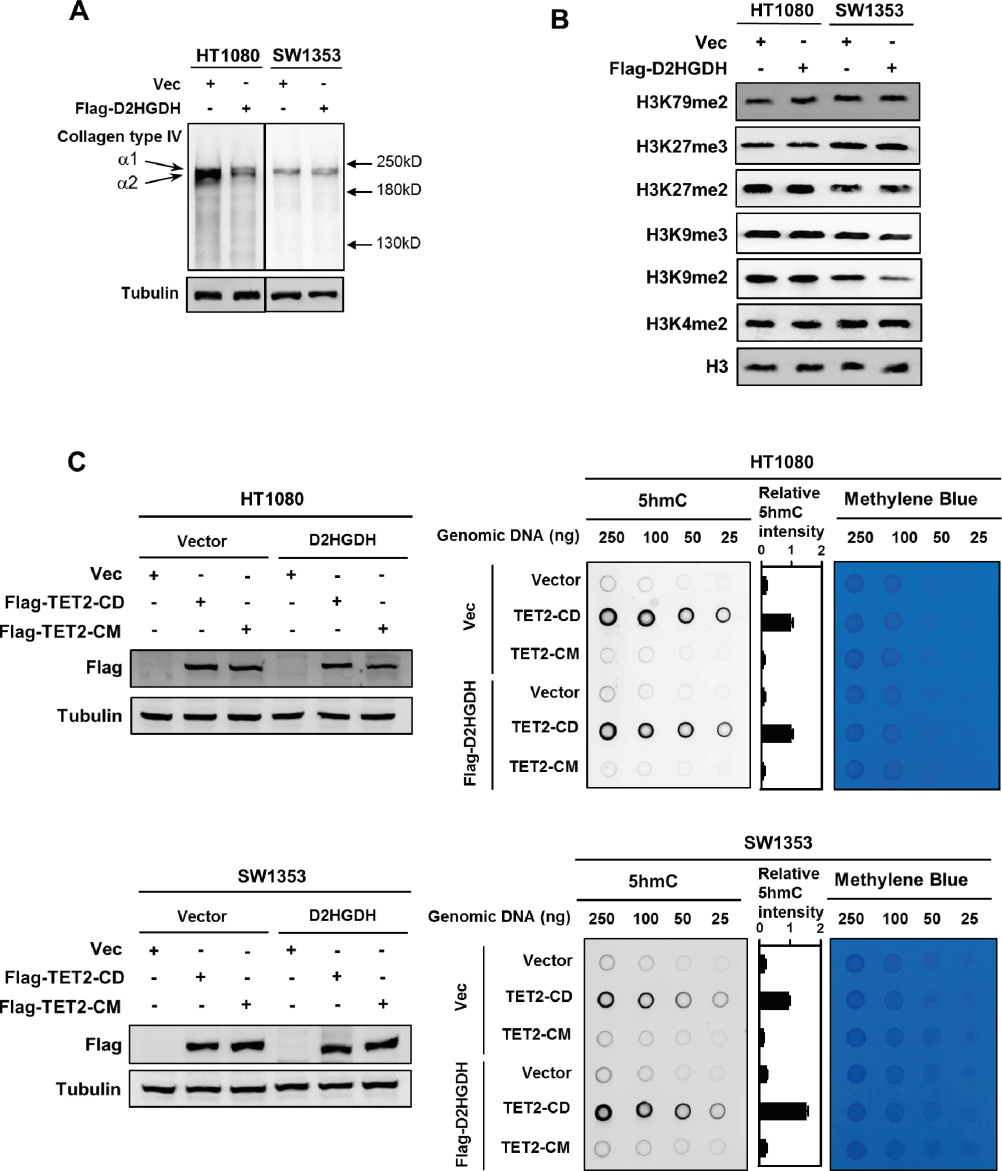
Ectopic expression of D2HGDH restores the activity of several α-KG-dependent dioxygenases **(A)** D2HGDH overexpression promotes the maturation of Collagen Type IV. Flag-tagged D2HGDH was stably overexpressed in HT1080 and SW1353 cells. In these stable cells, soluble collagen Type IV, which indicates the immature collagen, was detected by western blotting under reducing conditions with a collagen Type IV antibody. **(B)** D2HGDH overexpression decreases the levels of H3K9me2 and H3K9me3 in SW1353 cells, but not in HT1080 cells. Flag-tagged D2HGDH was stably overexpressed in HT1080 and SW1353 cells. Levels of various histone lysine methylations were detected by western blotting with specific antibodies. Total H3 was used as a loading control. **(C)** D2HGDH increases genomic 5 hmC levels in TET2 expressing cells. Flag-tagged D2HGDH was stably overexpressed in HT1080 and SW1353 cells, and these stable cells were transiently transfected with plasmids of wild-type TET2^CD^ or its catalytic inactive mutant TET2^CM^ as indicated. Forty-eight hours after transient transfection, genomic DNA was isolated, and 5 hmC was determined by dot-blot analyses. Methylene blue staining was performed to control equal DNA loading. The levels of transiently expressed Flag-tagged proteins were determined by western blot analysis (left panels).

It has previously been shown that D-2-HG can inhibit the hydroxylation and maturation of collagen type IV, leading to an increased level of soluble immature collagen type IV in brain specific IDH1-R132H knock-in mice [26]. We found that HT1080 cell produced more immature collagen type IV than SW1353 cells. Stable overexpression of D2HGDH significantly decreased the level of soluble collagen type IV in HT1080 cells, but not in SW1353 cells (Figure 2A), indicating that collagen hydroxylation in HT1080 cells is sensitive to D-2-HG. The reduction of immature collagen type IV by D2HGDH expression is consistent with an increased collagen hydroxylation activity.

D-2-HG is also known to inhibit the JmjC domain containing histone demethylases and the TET family 5mC hydroxylases, two potential targets of IDH1/2 mutations involved in epigenetic regulation [21]. We next investigated the effect of D-2-HG reduction by D2HGDH overexpression on histone and DNA methylation. We found that stable overexpression of D2HGDH in SW1353 cells preferentially decreased the levels of H3K9me2 and H3K9me3, two repressive histone methylation markers, whereas methylation of H3K4me2, H3K27me2, H3K27me3 or H3K79me2 was not significantly changed (Figure 2B). Notably, such alterations in histone methylation markers were, however, not observed in HT1080 cells after stable overexpression of D2HGDH (Figure 2B). It is worth noting that the D-2-HG levels in D2HGDH overexpressing HT1080 cells were much higher than D2HGDH overexpressing SW1353 cells (Figure 1B). A possible explanation is that although reduced, the remaining D-2HG in D2HGDH overexpressing HT1080 cells might still be sufficiently high to inhibit demethylation of H3K9me2 and H3K9me3.

The TET family of DNA hydroxylases catalyze three-sequential oxidation reactions of 5-methylcytosine (5mC) and play an essential role in active DNA demethylation [27]. 5-hydroxymethylcytosine (5 hmC) is the first hydroxylation intermediate generated from 5mC by TET enzymes. Genomic 5 hmC level in most cultured cells is normally very low, but it can be substantially increased by expression of the catalytic domain of TET2 (TET2^CD^) and thus can be easily detected by dot-blot using an antibody specifically recognizing 5 hmC [27–29]. We found that transient overexpression of TET2^CD^, but not the catalytic mutant TET2 (TET2^CM^), significantly increased genomic 5 hmC in both HT1080 and SW1353 cells (Figure 2C). Interestingly, transient overexpression of TET2^CD^ in SW1353 cells stably overexpressing D2HGDH led to higher levels of genomic 5 hmC than control cells (Figure 2C). Such a change in genomic 5 hmC was, however, not observed in HT1080 cells co-expressing TET2^CD^ and D2HGDH (Figure 2C). Collectively, our data suggest that D-2-HG reduction by D2HGDH overexpression can potentiate the activity of some histone demethylases and TET2 in the IDH2-mutated SW1353 cells. The lack of effect in HT1080 cells could be due to a less effective reduction of D-2-HG by D-2HGDH expression, and therefore the remaining D-2-HG might be still sufficient to inhibit the ectopically expressed TET2^CD^.

### Knockout of mutant IDH1-R132C allele eliminates D-2-HG and increases α-KG and its related metabolites

**Figure 3:**
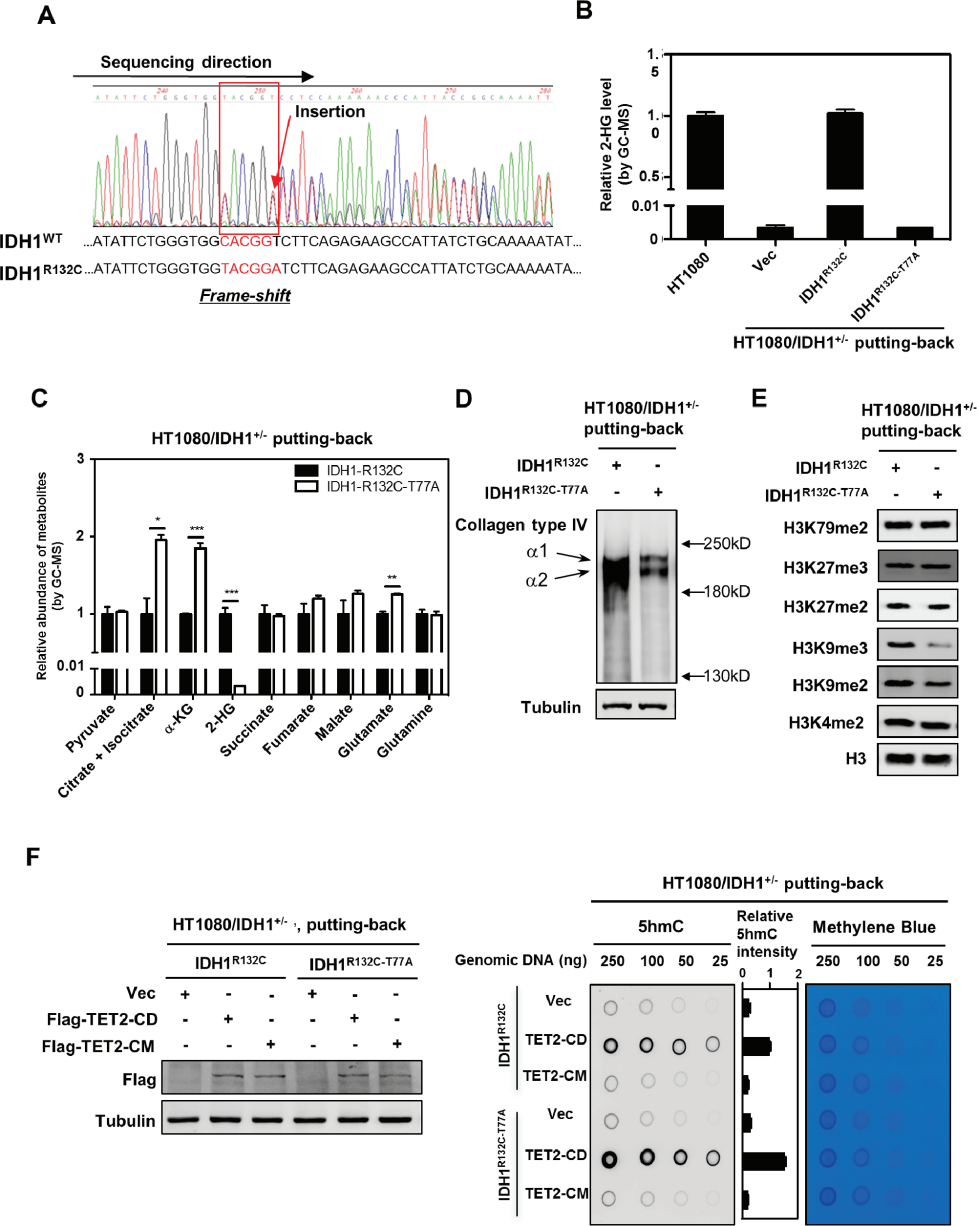
Putting-back IDH1-R132C in HT1080/IDH1^+/−^ cells restores D-2-HG and inhibits the activity of several α-KG-dependent dioxygenases **(A–B)** Deletion of the mutant IDH1-R132C allele in HT1080 cells eliminates D-2-HG production. The TALEN technology was applied to generate HT1080 cells with knock-out of endogenous IDH1-R132C (HT1080/IDH1^+/−^). Genomic sequencing confirmed the frame-shift mutation in IDH1-R132C allele, while the wild type IDH1 allele was intact (A). In accord, D-2-HG production was abolished in HT1080/IDH1^+/−^ cells as determined by GC-MS analysis (B). **(C)** Putting-back IDH1-R132C in HT1080/IDH1^+/−^ cells decreases the levels of α-KG and its neighboring metabolites. The intracellular concentrations of α-KG and other metabolites as indicated were determined by GC-MS analysis as described in “Method”. **(D)** Putting-back IDH1-R132C in HT1080/IDH1^+/−^ cells restores D-2-HG and inhibits the maturation of Collagen Type IV. Soluble immature collagen IV protein was detected by western blotting. **(E)** Putting-back IDH1-R132C in HT1080/IDH1^+/−^ cells increases the levels of H3K9me2 and H3K9me3. Histone lysine methylation was determined by western blotting with specific antibodies as indicated. Total H3 was used as a loading control. **(F)** Putting-back IDH1-R132C in HT1080/IDH1^+/−^ cells decreases genomic 5 hmC. In HT1080/IDH1^+/−^ cells re-expressing cells, wild-type TET2^CD^ or its catalytic inactive mutant TET2^CM^ was transiently expressed. Forty-eight hours after transfection, genomic DNA was isolated, and 5 hmC was determined by dot-blot analyses. Methylene blue staining was performed to control equal DNA loading. The levels of transiently expressed Flag-tagged proteins were determined by western blot analysis (left panel). For (B) and (C), shown are average values with standard error (SEM) of triplicated experiments. *denotes *p* < 0.05, **denotes *p* < 0.01, and ***denotes *p* < 0.001 for the indicated comparison.

As described above, D2HGDH overexpression had little effect on changing epigenetic markers in HT1080 cells. This promoted us to test whether the incapability of D2HGDH overexpression to affect the activity of histone and DNA demethylases in HT1080 cells is attributed to an incomplete clearance of D-2-HG. To achieve a complete D-2-HG clearance, we applied the TALEN technology to generate HT1080 cells with knock-out of endogenous IDH1-R132C (HT1080/IDH1^+/−^). After screening 213 clones, we isolated one clone with deletion of the mutant IDH1 R132C. In this clone, a frame-shift mutation in IDH1-R132C copy was confirmed by genomic sequencing (Figure 3A). As a result, IDH1 protein expression was decreased (Figure S6), and D-2-HG production was completely eliminated in HT1080/IDH1^+/−^ cells (Figures 3B and S5A and S5B).

To avoid the caveats of possible off-target effect of TALEN, we re-expressed mutant IDH1 in the HT1080/IDH1^+/−^ cells. As expected, re-introduction of IDH1-R132C mutant into HT1080/IDH1^+/−^ cells could restore D-2-HG to a level similar as the parental HT1080 cells (Figure 3B). As a negative control, re-expression of IDH1-R132C/T77A double mutant, which eliminated the D-2-HG producing activity, could not accumulate D-2-HG in HT1080/IDH1^+/−^ cells (Figure 3B).

Metabolic profiling analysis revealed that the α-KG level was significantly lower in HT1080/IDH1^+/−^ cells re-expressing IDH1-R132C mutant than cells re-expressing IDH1-R132C/T77A double mutant (decreased by 46%; *p* < 0.001) (Figure 3C). Additionally, intracellular levels of upstream metabolites of α-KG in the TCA cycle as well as certain downstream metabolites of α-KG in anaplerosis were also significantly decreased in HT1080/IDH1^+/−^ cells re-expressing IDH1-R132C mutant, including citrate/isocitrate (decreased by 49%; *p* < 0.05) and glutamate (decreased by 20%; *p* < 0.01) (Figure 3C).

Given that IDH1-R132C mutant uses NADPH as a co-factor to convert α-KG to D-2-HG, we next investigated whether IDH1-R132C mutation would affect cellular NADPH homeostasis. We found that there was no difference in the ratio of NADPH/NADP^+^ between HT1080/IDH1^+/−^ cells re-expressing IDH1-R132C mutant and those re-expressing IDH1-R132C/T77A double mutant (Figure S7A). In agreement, the ratio of GSH/GSSG was identical between the two cell lines (Figure S7B). Together, these data suggest that putting-back IDH1-R132C mutant in HT1080/IDH1^+/−^ cells does not affect cellular NADPH homeostasis and the redox potential although α-KG and its immediate neighboring metabolites are changed.

### Cellular D-2-HG suppresses the activity of several α-KG-dependent dioxygenases

We next studied whether putting-back IDH1-R132C mutant in HT1080/IDH1^+/−^ cells could affect the activity of α-KG-dependent dioxygenases. We examined the substrates of several α-KG dependent dioxygenases and found that re-introduction of IDH1-R132C mutant in HT1080/IDH1^+/−^ cells did not affect the protein levels of HIF-1α (Figure S8A and S8B) and Endostatin (Figure S9) when compared to those cells re-expressing IDH1-R132C/T77A double mutant. In contrast, putting-back IDH1-R132C mutant in HT1080/IDH1^+/−^ cells increased the level of soluble immature collagen type IV when compared to those cells re-expressing IDH1-R132C/T77A double mutant (Figure 3D). Furthermore, re-introduction of IDH1-R132C mutant in HT1080/IDH1^+/−^ cells increased the levels of H3K9me2 and H3K9me3 when compared to cells re-expressing IDH1-R132C/T77A double mutant (Figure 3E), indicating that IDH1-R132C mutant / D-2-HG can inhibit the activity of histone demethylases responsible for demethylation of H3K9me2/3. Additionally, the mutant IDH1 putting-back HT1080/IDH1^+/−^ cells transiently overexpressing TET2^CD^ exhibited lower levels of genomic 5 hmC than the IDH1-R132C/T77A double mutant expressing cells (Figure 3F), suggesting that IDH1-R132C mutant/D-2-HG can inhibit the activity of TET enzymes. Taken together, these findings suggest that D-2-HG in HT1080 cells plays a role in cellular regulation by inhibiting α-KG dependent dioxygenases, particularly those involved in collagen maturation, DNA demethylation, and H3K9 demethylation.

### D-2-HG accumulation is associated with selective regulation of gene expression in HT1080 cells

**Figure 4:**
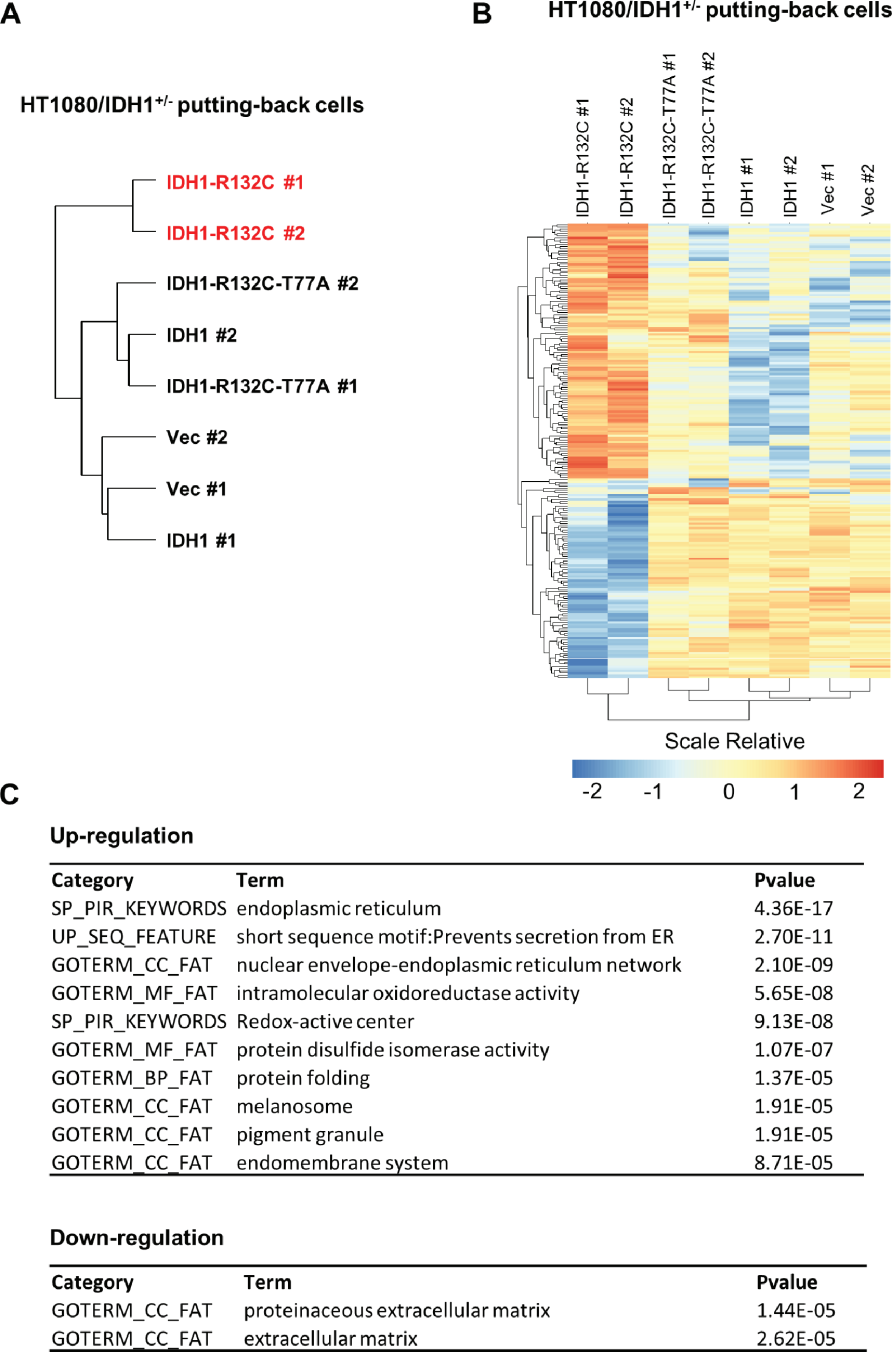
D-2-HG accumulation is associated with selective regulation of gene expression in HT1080 cells HT1080/IDH1^+/−^ cells putting-back empty vector (Vec), wild-type IDH1 (IDH1), D-2-HG producing related mutant IDH1 (IDH1-R132C) and enzymatic inactive double mutant IDH1 (IDH1-R132C-T77A) were used for gene expression profiling. Hierarchically clustering of cell lines based on gene expression are shown in **(A)**. The differentially expressed genes among the indicated clustered groups are presented in **(B)**. The functional annotation enrichment of up- and down-regulated pathways is listed in **(C)**.

*IDH* mutation has been reported to be associated with global hypermethylation (in both histone and CpG) and altered gene expression in acute myeloid leukemia (AML) and cholangiocarcinoma [30]. Mechanistically, D-2-HG produced by mutant IDH can alter epigenetic modifications by inhibiting α-KG-dependent histone and DNA demethylases [19]. These epigenetic changes may directly influence the chromatin status and transcription factor recruitment to modulate gene expression [31, 32]. As shown earlier in this study, D-2-HG is highly accumulated in HT1080/IDH1^+/−^ cells re-expressing IDH1-R132C mutant, but not in HT1080/IDH1^+/−^ cells re-expressing an empty vector, wild-type IDH1, or IDH1-R132C/T77A double mutant (Figure 3B). Importantly, RNA sequencing analysis revealed that HT1080/IDH1^+/−^ cells re-expressing IDH1-R132C mutant formed a cluster distinct from the other clusters containing HT1080/IDH1^+/−^ cells re-expressing an empty vector, wild-type IDH1, or IDH1-R132C/T77A double mutant (Figure 4A), further supporting the notion that D-2-HG accumulation is associated with changes in gene expression.

Surprisingly, there were only selective groups of genes whose transcriptional expression were different between HT1080/IDH1^+/−^ cells re-expressing IDH1-R132C mutant and the HT1080/IDH1^+/−^ cells re-expressing an empty vector, wild-type IDH1, or IDH1-R132C/T77A double mutant (Figure 4B). Interestingly, we detected significant up-regulation of genes involved in endoplasmic reticulum (ER), protein folding, and secretion in cells with D-2-HG production (Figure 4C). On the other hand, genes involved in extracellular matrix pathways are reduced. Although the detailed mechanism for these changes is unclear, our data suggest a potential role of D-2-HG in these cellular processes.

### D-2-HG does not affect cell proliferation but promotes anchorage independent growth *in vitro* and tumor growth *in vivo*

**Figure 5:**
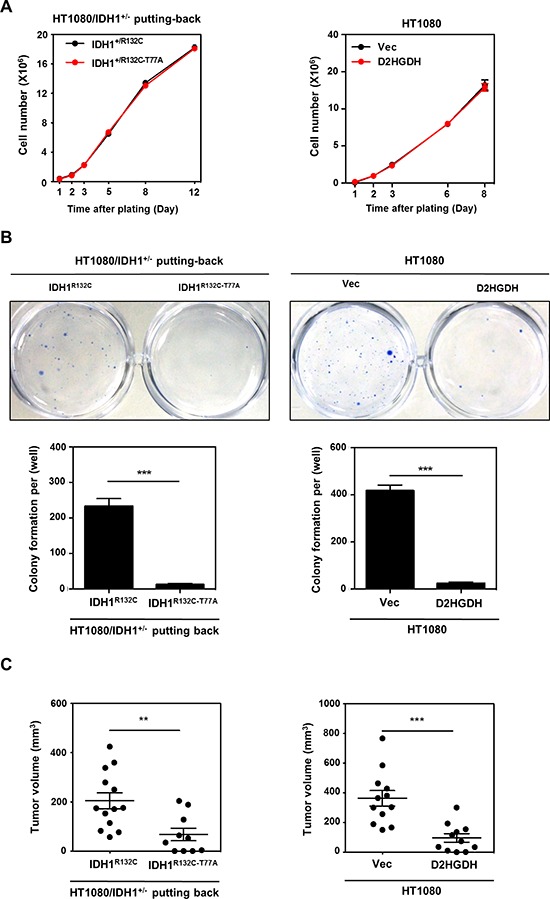
D-2-HG production is required for anchorage independent growth *in vitro* and tumor growth *in vivo* **(A)** D-2-HG does not affect cell proliferation. Growth curves of HT1080/IDH1^+/−^ cells with IDH1 putting-back (left panel) or HT1080 with D2GHDH overexpression (right panel) were determined. **(B)** D-2-HG promotes anchorage independent growth of HT1080 cells. HT1080/IDH1^+/−^ putting-back cells or HT1080 cell pools stably overexpressing Flag-D2HGDH were seeded in 6-well plates, and soft-agar colony formation assay was performed. Representative plates are shown. Quantification of colony numbers was derived from three independent experiments. **(C)** D-2-HG is required for tumor growth of HT1080 cells. HT1080/IDH1^+/−^ putting-back cells or HT1080 cell pools stably overexpressing Flag-D2HGDH (2*10^6^ cell) were injected subcutaneously into the flanks of nude mice. The tumor volume was carefully monitored during a period of 20–25 days after injection. In the end, tumors were extracted, photographed and weighted. Shown are average values with standard error (SEM) of triplicated experiments. **denotes *p* < 0.01 and ***denotes *p* < 0.001 for the indicated comparison.

By using genetically modified HT1080 cell lines described above, we examined the effect of D-2-HG on cell proliferation. Surprisingly, the rate of cell proliferation was identical between HT1080/IDH1^+/−^ cells re-expressing IDH1-R132C mutant and re-expressing IDH1-R132C/T77A double mutant (Figure 5A). It is possible that the epigenetic effect of D-2-HG accumulation on cell growth might be a slow process. Therefore, we had cultured the cell lines for over 20 passages, but still did not observe any obvious effect of D-2-HG accumulation on cell proliferation (data not show). Similarly, there was no difference in the rate of cell proliferation between stable HT1080 cells overexpressing D2HGDH and control cells expressing an empty vector (Figure 5A), further supporting the notion that D-2-HG does not directly impact cell proliferation, at least in HT1080 cells.

Next, we tested whether D-2-HG may affect cell migration. *In vitro* scratch assay revealed that there was no difference in cell migration between HT1080/IDH1^+/−^ cells re-expressing IDH1-R132C mutant and those re-expressing IDH1-R132C/T77A double mutant (Figure S10). Likewise, no visible difference was observed in cell motility during wound healing assays between HT1080 cells stably overexpressing D2HGDH and control cells expressing an empty vector (Figure S10). These results suggest that D-2-HG does not affect cell migration in HT1080 cells.

It is well established that anchorage-independent cell growth is a fundamental property of cancer cells. The ability of anchorage independent growth tightly correlates with tumorigenic and metastatic potentials *in vivo*. Strikingly, we found that re-introduction of IDH1-R132C mutant in HT1080/IDH1^+/−^ cells dramatically (*p* < 0.001) increased the cell's ability to exhibit anchorage independent growth compared to those re-expressing IDH1-R132C/T77A double mutant (Figure 5B). Moreover, D2HDGH overexpression also reduced the ability of HT1080 cells to exhibit anchorage independent cell growth (*p* < 0.001) (Figure 5B), even though the reduction of D-2-HG in these cells was incomplete (Figure 1B). These data suggest that D-2-HG plays an important role in promoting anchorage-independent cell growth in HT1080 cells.

The effect of D-2-HG on stimulating anchorage-independent cell growth led us to test whether D-2-HG renders growth advantage to tumor cells *in vivo*. We performed xenograft experiments using HT1080/IDH1^+/−^ cells re-expressing IDH1-R132C mutant or those re-expressing IDH1-R132C/T77A double mutant. Tumor growth was monitored over a period of 20–25 days. Our data demonstrated that the tumor volumes in nude mice injected with HT1080/IDH1^+/−^ cells re-expressing IDH1-R132C were significantly larger than those from mice injected with HT1080/IDH1^+/−^ cells re-expressing IDH1-R132C/T77A double mutant (Tumor size at 25 days: 204 ± 33 mm^3^ vs. 67 ± 25 mm^3^ for IDH1-R132C vs. IDH1-R132C/T77A; *p* = 0.0045) (Figure 5C). Finally, we performed similar xenograft experiments using stable HT1080 cells with or without ectopic expression of D2HGDH. Again, tumor cell growth was dramatically suppressed by the expression of D2HGDH (Tumor size at 25 days: 343 ± 52 mm^3^ vs. 76 ± 22 mm^3^ for D2HGDH vs. Vec; p = 0.0002) (Figure 5C). Collectively, these data support a notion that D-2-HG promotes the tumorigenicity of HT1080 cells *in vivo*. Furthermore, even a three-fold reduction of D-2-HG was sufficient to suppress tumor growth of IDH1-mutated HT1080 cells.

## DISCUSSION

Mutations in IDH1 or IDH2 are frequently observed in human cancers, particularly with high frequency in secondary glioma, acute myeloid leukemia, and chondrosarcoma [2–4]. The exact mechanism regarding how IDH mutations promote tumorigenesis is not fully understood, because much of previous mechanistic studies have been heavily relying on overexpression or knock-in of mutant IDH in tumor cell lines that naturally do not have native IDH mutations, and their cancer growth property may not depend on the ectopically expressed mutant IDH1/2. It is therefore difficult to ascertain the role of IDH mutations in tumorigenesis. In this report, we have used sarcoma cell lines HT1080 and SW1353 that contain endogenous mutations of IDH1/2 to investigate the function of IDH mutation in tumorigenesis. Previous studies have suggested that the oncometabolite D-2-HG produced by cancer-associated mutant IDH1/2 may convey oncogenic signals through altering epigenetic modifications, which leads to altered gene expression and growth promotion [19, 20, 33, 34]. We manipulated D-2-HG levels in HT1080 and SW1353 cells by either stable overexpression of D2HGDH or knocking-out of the endogenous mutant *IDH1* gene.

Altered epigenetic modifications, such as histone and DNA methylation, can influence chromatin structure and help to recruit transcription factors and other regulatory proteins to the DNA, thereby regulating gene expression [31, 32]. Supporting this notion, IDH mutation has been shown to impair KDM4C/JMJD2C, which is responsible for demethylation of the repressive histone H3K9 di- and trimethylation, alteration of cell lineage gene expression, and differentiation arrest in 3T3-L1 cells [35]. Moreover, *in vitro* enzymatic studies have revealed that JMJD2A and JMJD2C, two histone H3K9me2/3 demethylases, are among the enzymes examined most sensitive to inhibition by D-2-HG, with IC_50_ of 24 ± 2 μM and 79 ± 7 μM, respectively [20]. Here, we show that elimination of D-2-HG results in the most dramatic effect on H3K9 methylation in HT1080 and SW1353 cells, while methylation status of other histone lysine residues is not significantly altered. These findings indicate that KDM4C/JMJD2C is inhibited by D-2-HG accumulated in different cancer cells, and that the other histone demethylases maybe more resistant to inhibition by D-2-HG. In addition, we also observed that the TET2-catalyzed 5 hmC production is inhibited by D-2-HG accumulated in HT1080 and SW1353 cells. A partial clearance of D-2-HG by D2HGDH overexpression in HT1080 cells is not sufficient to relieve the inhibition on TET2-catalyzed 5 hmC production, indicating that TET2 is rather sensitive to D-2-HG inhibition. Taken together, our data suggest that D-2-HG accumulated in cancer cells inhibits enzymes involved in DNA and histone demethylation with high selectivity.

Our results show that D-2-HG plays no direct role in cell proliferation in routine laboratory culture plates. Notably, ectopic expression of D2HGDH which partially reduces D-2-HG, does not change histone methylation markers and TET2-induced 5 hmC production in HT1080 cells, yet D2HGDH overexpression effectively inhibits the anchorage independent growth and tumor growth of these cells. Therefore, the oncogenic role of D-2-HG in promoting anchorage-independent cell growth and tumorigenicity may be more complex than expected. Whether other α-KG-dependent dioxygenases besides KDMs and TETs would contribute to the oncogenic function of D-2-HG requires further investigation. Our observations that D-2-HG is required for tumor growth of cancer cells with IDH1/2 mutations suggest that inhibition of mutant IDH1/2 could be an attractive strategy for therapeutic treatment of cancers with IDH mutations. Consistently, inhibition of mutant IDH1 was recently reported to have preclinical efficacy in IDH1 mutant glioma cells and IDH2 mutant AML. In the present study, we show that D2HGDH overexpression can reverse the oncogenic effects of IDH1/2 mutation. Therefore, induction of D2HGDH expression may be another potential approach to treat IDH-mutated cancer. Future efforts will thus be needed to investigate the molecular mechanism of D2HGDH regulation and its potential applications as a therapeutic strategy for D-2-HG lowering in *IDH*-mutated tumors.

## MATERIALS AND METHODS

### Antibodies

Antibodies against D2HGDH (Proteintech, 13895–1-AP), L2HGDH (Proteintech, 15707–1-AP), Flag (Sigma-Aldrich, F1804), Tubulin (Neomarker, MS-581-P1), β-actin (Genescript, A00702), IDH1 (Epitomics, 8057–1), H3 (CST, 4499), H3K4me2 (Millipore, 07–030), H3K9me2 (Abcam, ab1220), H3K9me3 (Abcam, ab8898), H3K27me2 (Abcam, ab1298), H3K27me3 (Millipore, 17–622), H3K79me2 (Abcam, ab3594), 5 hmC (Active Motif, 39769), Collagen Type IV (Rockland 600–401-106), HIF-1α (BD, 610958) and Endostatin (Abcam, ab64569) were purchased commercially.

### Plasmid construction

Full-length cDNAs of human D2HGDH, L2HGDH, and IDH1 were amplified by PCR and then sub-cloned into pBABEpuro or pQCXIH vector for retroviral infection. Human TET2 cDNA was sub-cloned into pcDNA3 for transient transfection. Point mutations of the indicated constructions were generated by standard site-directed mutagenesis techniques. All constructions were confirmed by direct sequencing before further applications.

### Cell culture, transfection and stable cell line generation

Two sacoma cell lines, HT1080 and SW1353, were maintained in Minimum Essential Medium (MEM; Gibco) and L-15 medium (Leibovitz), respectively. Both medium were supplemented with 10% fetal bovine serum (Biological Industries). HEK293T cells were cultured in Dulbecco's Modified Eagle's Medium (DMEM; Gibco) supplement with 10% newborn calf serum (Biochrom).

Plasmid transfection was carried out by polyethylenimine (PEI; Polysciences) method, according to the manufacturer's protocol.

Cells stably expressing the indicated proteins were established by standard retroviral infection, and selected in 2 μg/ml puromycin (Ameresco) or 50 μg/ml hygromycin B (Ameresco) for 7 days.

### Generation of HT1080/IDH1^+/−^ cells by TALEN technology

The TALEN target site within the *IDH1* gene was selected manually and was further confirmed by sequencing to exclude polymorphism. The units corresponding to 5′-tACGAAATATTCTGGGt-3′ was assembled and sub-cloned into pCS2-PEAS vector as the left TALE binding site, while the units corresponding to 5′-tGCAGATAATGGCTTCt-3′ was assembled and sub-cloned into pCS2-PERR vector as the right TALE binding site.

HT1080 cells were transfected by the indicated TALE constructs. 48 hours after transfection, the cells were diluted and seeded into 96-well plates. Mono-clones were marked and grew for verification. To further confirm the modification of endogenous *IDH1* gene, genomic DNA was extracted using standard phenol/chloroform extraction method and the targeted region was amplified and sequenced. Primers used for monitoring the TALE editing region were IDH1-T1-F, 5′-CCATCACTGCAGTTGTAGGTT-3′ and IDH1-T1-R, 5′-CCAGAAATTTCCAACTTGTATGTGA-3′F.

### Western blotting

Cell were lysed in NP-40 buffer containing 150 mM sodium chloride, 50 mM Tris at pH 7.4, 0.3% (v/v) NP-40 and a cocktail of protease inhibitors or lysed directly in SDS-PAGE loading buffer. For collagen Type IV protein detection, cultured cells were homogenized in a buffer containing 200 mM sodium chloride, 20 mM Tris at pH 7.4, 100 mM glycine, 0.1% (v/v) Triton X-100, 50 μM DTT and a cocktail of protease inhibitor. Western blotting was performed according to standard protocol.

### Metabolite extraction and GC-MS analysis

Post-confluent cells in 6-well plates were homogenized in 0.5 ml chilled 80% (v/v) methanol containing heptadecanoic acid (Sigma, H3500) as an internal standard to monitor batch reproducibility. The samples were centrifuged at 12, 000 rpm for 10 min and the supernatant was transferred to a high recovery glass sampling vial (CNW, VAAP-31509–1232-100) to vacuum dry at room temperature. The residue was oximated with 30 μl pyridine containing 20 mg/ml methoxyamine hydrochloride (Sigma-Aldrich, 226904) at 37°C overnight and further derivatized with 20 μl N-tert-Butyldimethylsilyl-N-methyltrifluoroacetamide (Sigma-Aldrich, 394882) at 70°C for 30 min. 1 μl aliquot of the derivatized sample was injected into Aligent 7890A gas chromatography coupled with Agilent 5975C mass spectrometer. Separation was achieved on a HP-5ms fused-silica capillary column (30 m × 250 μm i.d.; 5% diphenyl - 95% emthylpolysiloxane bonded and crosslinked) with helium as the carrier gas at a constant flow rate of 1 ml/min through the column. The temperature of front injection, MSD transfer interface and electron impact (EI) ion source were set to 280°C, 290°C and 230°C, respectively. The GC oven temperature was set to 100°C for 3 min, with an increment rate of 10°C/min to 140°C, 8°C/min to 260°C, 10°C/min to 310°C and a final 5 min maintenance at 310°C. The electron impact ionization was 70 eV. After 5 min of solvent delay, the mass data was collected at full scan mode (m/z 50–600).

### Measurement of intracellular NADPH and NADP^+^ levels

The intracellular levels of NADPH and NADP+ were measured by enzymatic cycling methods as previously described [36, 37]. In brief, 1.8 × 10^6^ cells were seeded in 10 cm dishes. On the next day, cells were lysed in 400 μL of extraction buffer (20 mM NAM, 20 mM NaHCO3, 100 mM Na_2_CO_3_) and centrifuged at 1, 200g for 15 min. For NADPH extraction, 150 μL of the supernatant was incubated in a heating block for 30 min at 60°C. 160 μL of NADP-cycling buffer (100 mM Tris-HCl, pH8.0; 0.5 mM thiazolylblue; 2 mM phenazine ethosulfate; 5 mM EDTA) containing 1.3U of G6PD was added to a 96-well plate containing 20 μL of the cell extract. After incubation for 1 min at 30°C in darkness, 20 μL of 10 mM G6P was added to the mixture, and the change in absorbance at 570 nm was measured every 30 sec for 10 min at 30°C by the SpectraMax M5 Microplate Reader (Molecular Devices). All the samples were run in triplicate. The concentration of NADP^+^ was calculated by subtracting NADPH (heated sample) from total NADP + NADPH (unheated sample).

### Measurement of intracellular GSSG and GSH levels

Intracellular GSSG and GSH levels were determined by using liquid chromatography–mass spectrometry (LC-MS/MS). In short, cells were harvested by using 80% (v/v) pre-cooling (−80°C) methanol and centrifuged at 4°C at 12000 rpm for 15 min. The supernatants were then subjected to LC-MS analysis using a ShimazuLC (LC-20AB pump) system coupled with 4000 qtrap triple-quadrupole mass spectrometer (AB sciex). A Phenomenex NH2 column (50mm × 2.0mm I.D., 5 μm particle size, 100Aº) was used. The mass spectrometer was optimized and set up in selected reaction monitoring (SRM) scan mode for monitoring the M-H of GSSG (m/z 611.6→306.2) and GSH (m/z 306.2→142.8). The Analyst Software was used for analysis.

### DNA dot-blot assay

Genomic DNA was extracted using phenol chloroform method and quantified using NanoDrop. DNA was serial diluted, spotted on a nitrocellulose membrane and crosslinked with the membrane under ultraviolet lamp. The membrane was blocked in 5% milk and then for subsequent primary and secondary antibodies incubation. The membrane was scanned with a Typhoon scanner (GE Healthcare). The 5 hmC intensity was quantified by Image Quanta software (GE Healthcare).

### Gene expression analysis

Next-generation sequencing was performed with standard method. Briefly, total RNA was extracted using TransZol Up (TransGen). Samples were quantified with Agilent 2100 Bioanalyzer. The library preparation and RNA sequencing was conducted using Illumina TruSeq and Illumina HiSeq 2500 platform (Illumina Inc., San Diego, CA).

For sequencing result analyais, after quality filtering, paired-end reads were aligned to the human reference genome (UCSC hg19) by TopHat software (version 2.09) with default parameters except that Ensemble GTF gene annotation (-G) (version 63) was used to guide the junction reads aligment. Read number counting and differentially expressed gene calling were carried out by Htseq-count scripts and limma package. Three comparisons (i.e. HT1080/IDH1^+/−^ cell putting-back IDH1-R132C vs. IDH1-R132C-T77A, IDH1-R132C vs. IDH1, and IDH1-R132C vs. vector) were performed and differentially expressed genes were filtered with *p*-value less than 0.001. Functional annotation enrichment analysis was done by using the DAVID website.

### Soft-ager colony formation assay

1.5 ml medium containing 0.5% agar was poured into 6-well plates at first and acted as bottom layer to segregate the cells from touching with the surface of plate bottom. After solidifying, a top layer medium containing 0.3% agar and 3000 cells was poured. The plates were placed in the incubator and the medium was changed every 3 days for 30 days. Then the colonies were fixed by 4% paraformaldehyde in PBS and stained with 0.005% crystal violet solution. The colonies larger than 100 μm were counted per well. Every group was completed in biological triplicate.

### Cell proliferation and xenograft studies

Stable HT1080 cells (3 × 10^4^) were seeded in triplicate in 6-well plates. The cell number was counted at the indicated time points.

Nude mice (male, 4- to 6-week old) were injected subcutaneously with stable HT1080 cells (2 × 10^6^). The tumor volume was measured each week. The tumors were harvested and measured at the indicated time points.

### Statistical methods

Results were presented as mean ± SEM unless otherwise specified. Statistical analysis is performed using a two-tailed unpaired Student's *t*-test. The *P* values less than 0.05 were considered significant (* < 0.05, ** < 0.01, *** < 0.0001).

## SUPPLEMENTARY FIGURES



## References

[1] Warburg O (1956). On the origin of cancer cells. Science.

[2] Mardis ER, Ding L, Dooling DJ, Larson DE, McLellan MD, Chen K, Koboldt DC, Fulton RS, Delehaunty KD, McGrath SD, Fulton LA, Locke DP, Magrini VJ, Abbott RM, Vickery TL, Reed JS (2009). Recurring mutations found by sequencing an acute myeloid leukemia genome. N Engl J Med.

[3] Yan H, Parsons DW, Jin G, McLendon R, Rasheed BA, Yuan W, Kos I, Batinic-Haberle I, Jones S, Riggins GJ, Friedman H, Friedman A, Reardon D, Herndon J, Kinzler KW, Velculescu VE (2009). IDH1 and IDH2 mutations in gliomas. N Engl J Med.

[4] Amary MF, Bacsi K, Maggiani F, Damato S, Halai D, Berisha F, Pollock R, O'Donnell P, Grigoriadis A, Diss T, Eskandarpour M, Presneau N, Hogendoorn PC, Futreal A, Tirabosco R, Flanagan AM (2011). IDH1 and IDH2 mutations are frequent events in central chondrosarcoma and central and periosteal chondromas but not in other mesenchymal tumours. The Journal of pathology.

[5] Cairns RA, Iqbal J, Lemonnier F, Kucuk C, de Leval L, Jais JP, Parrens M, Martin A, Xerri L, Brousset P, Chan LC, Chan WC, Gaulard P, Mak TW (2012). IDH2 mutations are frequent in angioimmunoblastic T-cell lymphoma. Blood.

[6] Wang P, Dong Q, Zhang C, Kuan PF, Liu Y, Jeck WR, Andersen JB, Jiang W, Savich GL, Tan TX, Auman JT, Hoskins JM, Misher AD, Moser CD, Yourstone SM, Kim JW (2013). Mutations in isocitrate dehydrogenase 1 and 2 occur frequently in intrahepatic cholangiocarcinomas and share hypermethylation targets with glioblastomas. Oncogene.

[7] Dang L, White DW, Gross S, Bennett BD, Bittinger MA, Driggers EM, Fantin VR, Jang HG, Jin S, Keenan MC, Marks KM, Prins RM, Ward PS, Yen KE, Liau LM, Rabinowitz JD (2009). Cancer-associated IDH1 mutations produce 2-hydroxyglutarate. Nature.

[8] Ward PS, Patel J, Wise DR, Abdel-Wahab O, Bennett BD, Coller HA, Cross JR, Fantin VR, Hedvat CV, Perl AE, Rabinowitz JD, Carroll M, Su SM, Sharp KA, Levine RL, Thompson CB (2010). The common feature of leukemia-associated IDH1 and IDH2 mutations is a neomorphic enzyme activity converting alpha-ketoglutarate to 2-hydroxyglutarate. Cancer cell.

[9] Andersson AK, Miller DW, Lynch JA, Lemoff AS, Cai Z, Pounds SB, Radtke I, Yan B, Schuetz JD, Rubnitz JE, Ribeiro RC, Raimondi SC, Zhang J, Mullighan CG, Shurtleff SA, Schulman BA (2011). IDH1 and IDH2 mutations in pediatric acute leukemia. Leukemia.

[10] Gross S, Cairns RA, Minden MD, Driggers EM, Bittinger MA, Jang HG, Sasaki M, Jin S, Schenkein DP, Su SM, Dang L, Fantin VR, Mak TW (2010). Cancer-associated metabolite 2-hydroxyglutarate accumulates in acute myelogenous leukemia with isocitrate dehydrogenase 1 and 2 mutations. The Journal of experimental medicine.

[11] Andronesi OC, Rapalino O, Gerstner E, Chi A, Batchelor TT, Cahill DP, Sorensen AG, Rosen BR (2013). Detection of oncogenic IDH1 mutations using magnetic resonance spectroscopy of 2-hydroxyglutarate. The Journal of clinical investigation.

[12] Choi C, Ganji SK, DeBerardinis RJ, Hatanpaa KJ, Rakheja D, Kovacs Z, Yang XL, Mashimo T, Raisanen JM, Marin-Valencia I, Pascual JM, Madden CJ, Mickey BE, Malloy CR, Bachoo RM, Maher EA (2012). 2-hydroxyglutarate detection by magnetic resonance spectroscopy in IDH-mutated patients with gliomas. Nat Med.

[13] Kranendijk M, Struys EA, Salomons GS, Van der Knaap MS, Jakobs C (2012). Progress in understanding 2-hydroxyglutaric acidurias. Journal of inherited metabolic disease.

[14] Achouri Y, Noel G, Vertommen D, Rider MH, Veiga-Da-Cunha M, Van Schaftingen E (2004). Identification of a dehydrogenase acting on D-2-hydroxyglutarate rate. Biochemical Journal.

[15] Rzem R, Veiga-da-Cunha M, Noel G, Goffette S, Nassogne MC, Tabarki B, Scholler C, Marquardt T, Vikkula M, Van Schaftingen E (2004). A gene encoding a putative FAD-dependent L-2-hydroxyglutarate dehydrogenase is mutated in L-2-hydroxyglutaric aciduria. Proceedings of the National Academy of Sciences of the United States of America.

[16] Topcu M, Jobard F, Halliez S, Coskun T, Yalcinkayal C, Gerceker FO, Wanders RJ, Prud'homme JF, Lathrop M, Ozguc M, Fischer J (2004). L-2-Hydroxyglutaric aciduria: identification of a mutant gene C14orf160, localized on chromosome 14q22.1. Human molecular genetics.

[17] Kranendijk M, Struys EA, Salomons GS, Van der Knaap MS, Jakobs C (2012). Progress in understanding 2-hydroxyglutaric acidurias. Journal of inherited metabolic disease.

[18] Reitman ZJ, Sinenko SA, Spana EP, Yan H (2014). Genetic dissection of leukemia-associated IDH1 and IDH2 mutants and 2-hydroxyglutarate in Drosophila. Blood.

[19] Xu W, Yang H, Liu Y, Yang Y, Wang P, Kim SH, Ito S, Yang C, Wang P, Xiao MT, Liu LX, Jiang WQ, Liu J, Zhang JY, Wang B, Frye S (2011). Oncometabolite 2-hydroxyglutarate is a competitive inhibitor of alpha-ketoglutarate-dependent dioxygenases. Cancer cell.

[20] Chowdhury R, Yeoh KK, Tian YM, Hillringhaus L, Bagg EA, Rose NR, Leung IK, Li XS, Woon EC, Yang M, McDonough MA, King ON, Clifton IJ, Klose RJ, Claridge TD, Ratcliffe PJ (2011). The oncometabolite 2-hydroxyglutarate inhibits histone lysine demethylases. EMBO Rep.

[21] Yang H, Ye D, Guan KL, Xiong Y (2012). IDH1 and IDH2 mutations in tumorigenesis: mechanistic insights and clinical perspectives. Clinical cancer research: an official journal of the American Association for Cancer Research.

[22] Losman JA, Looper RE, Koivunen P, Lee S, Schneider RK, McMahon C, Cowley GS, Root DE, Ebert BL, Kaelin WG (2013). (R)-2-hydroxyglutarate is sufficient to promote leukemogenesis and its effects are reversible. Science.

[23] Saha SK, Parachoniak CA, Ghanta KS, Fitamant J, Ross KN, Najem MS, Gurumurthy S, Akbay EA, Sia D, Cornella H, Miltiadous O, Walesky C, Deshpande V, Zhu AX, Hezel AF, Yen KE (2014). Mutant IDH inhibits HNF-4alpha to block hepatocyte differentiation and promote biliary cancer. Nature.

[24] Loenarz C, Schofield C (2008). Expanding chemical biology of 2-oxoglutarate oxygenases. Nature Chemical Biology.

[25] Hirsila M, Koivunen P Fau - Gunzler V, Gunzler V Fau - Kivirikko KI, Kivirikko Ki Fau - Myllyharju J, Myllyharju J (2003). Characterization of the human prolyl 4-hydroxylases that modify the. The Journal of biological chemistry.

[26] Sasaki M, Knobbe CB, Itsumi M, Elia AJ, Harris IS, Chio IIC, Cairns RA, McCracken S, Wakeham A, Haight J (2012). D-2-hydroxyglutarate produced by mutant IDH1 perturbs collagen maturation and basement membrane function. Genes & development.

[27] He YF, Li BZ, Li Z, Liu P, Wang Y, Tang Q, Ding J, Jia Y, Chen Z, Li L, Sun Y, Li X, Dai Q, Song CX, Zhang K, He C (2011). Tet-mediated formation of 5-carboxylcytosine and its excision by TDG in mammalian DNA. Science.

[28] Tahiliani M, Koh KP, Shen Y, Pastor WA, Bandukwala H, Brudno Y, Agarwal S, Iyer LM, Liu DR, Aravind L, Rao A (2009). Conversion of 5-methylcytosine to 5-hydroxymethylcytosine in mammalian DNA by MLL partner TET1. Science.

[29] Ito S, D'Alessio AC, Taranova OV, Hong K, Sowers LC, Zhang Y (2010). Role of Tet proteins in 5mC to 5 hmC conversion, ES-cell self-renewal and inner cell mass specification. Nature.

[30] Figueroa ME, Abdel-Wahab O, Lu C, Ward PS, Patel J, Shih A, Li Y, Bhagwat N, Vasanthakumar A, Fernandez HF (2010). Leukemic IDH1 and IDH2 mutations result in a hypermethylation phenotype, disrupt TET2 function, and impair hematopoietic differentiation. Cancer cell.

[31] Bell O, Tiwari VK, Thoma NH, Schubeler D (2011). Determinants and dynamics of genome accessibility. Nature reviews Genetics.

[32] Musselman CA, Lalonde ME, Cote J, Kutateladze TG (2012). Perceiving the epigenetic landscape through histone readers. Nature structural & molecular biology.

[33] Figueroa ME, Abdel-Wahab O, Lu C, Ward PS, Patel J, Shih A, Li Y, Bhagwat N, Vasanthakumar A, Fernandez HF, Tallman MS, Sun Z, Wolniak K, Peeters JK, Liu W, Choe SE (2010). Leukemic IDH1 and IDH2 mutations result in a hypermethylation phenotype, disrupt TET2 function, and impair hematopoietic differentiation. Cancer cell.

[34] Turcan S, Rohle D, Goenka A, Walsh LA, Fang F, Yilmaz E, Campos C, Fabius AW, Lu C, Ward PS, Thompson CB, Kaufman A, Guryanova O, Levine R, Heguy A, Viale A (2012). IDH1 mutation is sufficient to establish the glioma hypermethylator phenotype. Nature.

[35] Lu C, Ward PS, Kapoor GS, Rohle D, Turcan S, Abdel-Wahab O, Edwards CR, Khanin R, Figueroa ME, Melnick A, Wellen KE, O'Rourke DM, Berger SL, Chan TA, Levine RL, Mellinghoff IK (2012). IDH mutation impairs histone demethylation and results in a block to cell differentiation. Nature.

[36] Zerez CR, Moul DE, Gomez EG, Lopez VM, Andreoli AJ (1987). Negative modulation of Escherichia coli NAD kinase by NADPH and NADH. J Bacteriol.

[37] Wagner TC, Scott MD (1994). Single extraction method for the spectrophotometric quantification of oxidized and reduced pyridine nucleotides in erythrocytes. Anal Biochem.

