# Supplementary material for: D-2-hydroxyglutarate is essential for maintaining oncogenic property of mutant IDH-containing cancer cells but dispensable for cell growth
Source: Oncotarget. 2015 Mar 25;6(11):8606–20. doi: 10.18632/oncotarget.3330 (PMC4496170; doi:10.18632/oncotarget.3330)
Supplement: Supplementary file 1 [file oncotarget-06-8606-s001.pdf]

## SUPPLEMENTARY FIGURES

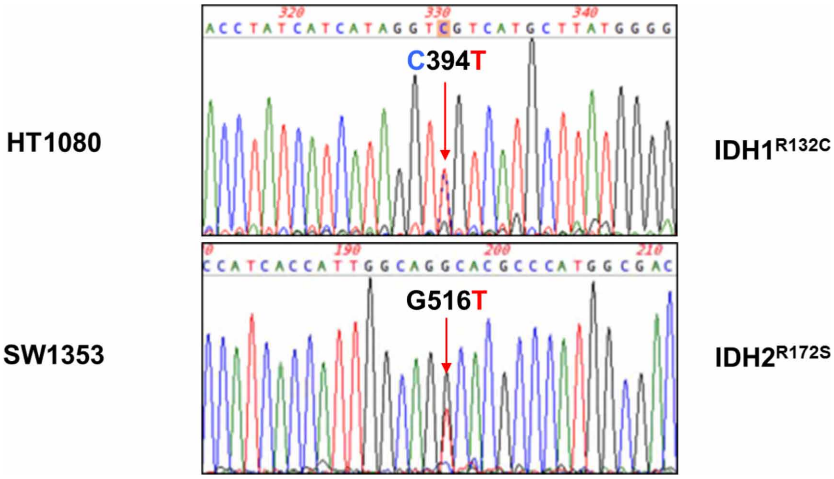


**Supplementary Figure S1: HT1080 and SW1353 cells harbor heterozygous IDH1-R132C and IDH2-R172S mutations, respectively.** Sequencing confirmed the heterozygous mutation sites of IDH1 C394T and IDH2 G516T in HT1080 and SW1353 cells, respectively.

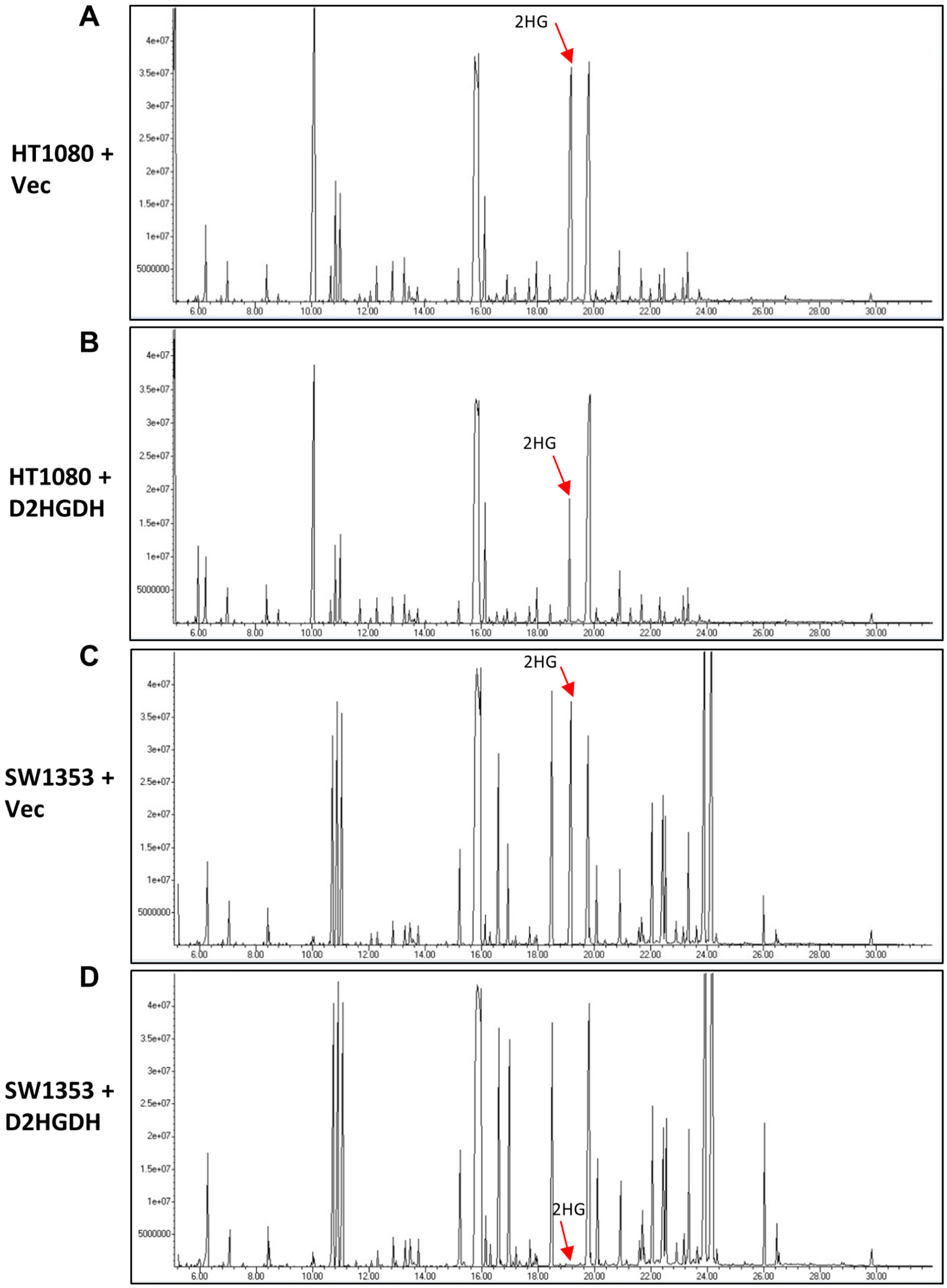


**Supplementary Figure S2: Ectopic expression of D2HGDH reduces D-2-HG level in IDH-mutated cells.** The intracellular level of 2-HG in parental HT1080 and SW1353 cells and stable HT1080 and SW1353 cells overexpressing Flag-tagged D2HGDH was determined by GC-MS analysis. 2-HG peak was firstly analyzed by searching for and matching the NIST database and was further confirmed by D-2-HG standard. The quantification was done using the main fragment m/z 433.

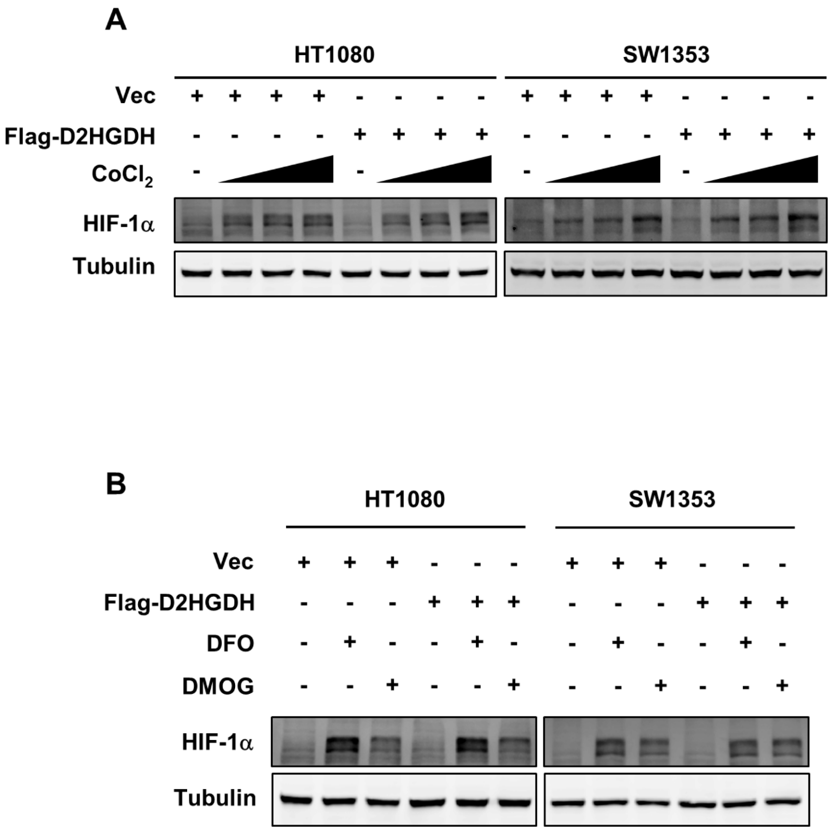


**Supplementary Figure S3: Ectopic expression of D2HGDH does not affect HIF-1α protein levels in IDH-mutated cells.** Flag-tagged D2HGDH was stably overexpressed in HT1080 and SW1353 cells, and these cells were treated without or with $CoCl_2$ (200 µµm) **(A)**, DFO (0.5 mM) **(B)**, DMOG (1 mM) for 6 hours. (B). The protein level of HIF-1 was determined by western blotting. Tubulin was detected as a loading control.

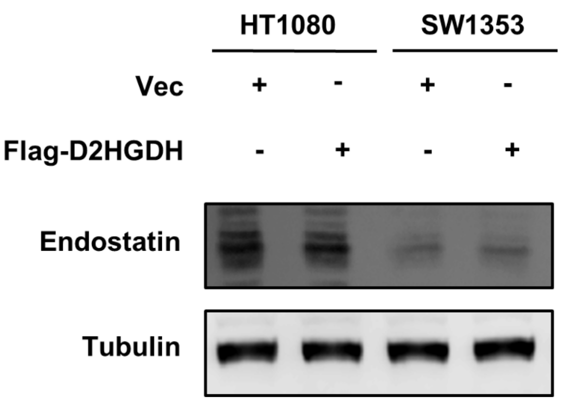


**Supplementary Figure S4: Ectopic expression of D2HGDH does not affect Endostatin protein levels in IDH-mutated cells.** Flag-tagged D2HGDH was stably overexpressed in HT1080 and SW1353 cells, and the protein level of Endostatin was detected by western blotting. Tubulin was detected as a loading control.

**A**

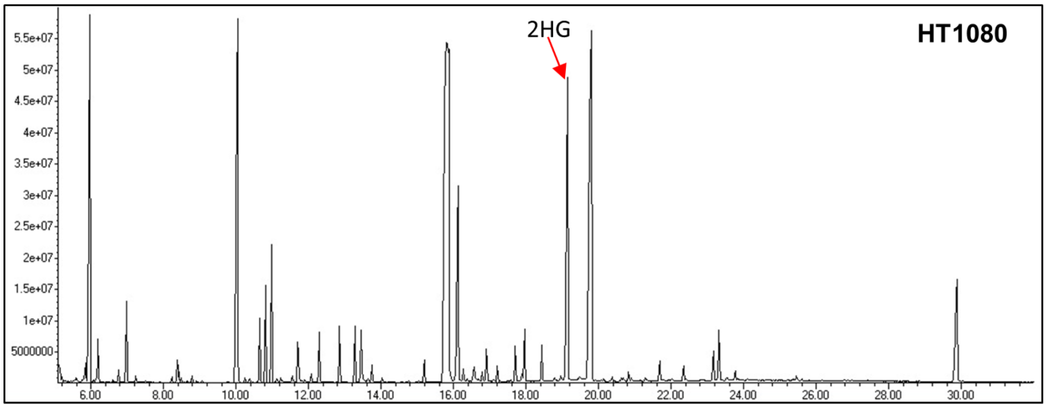


**B**

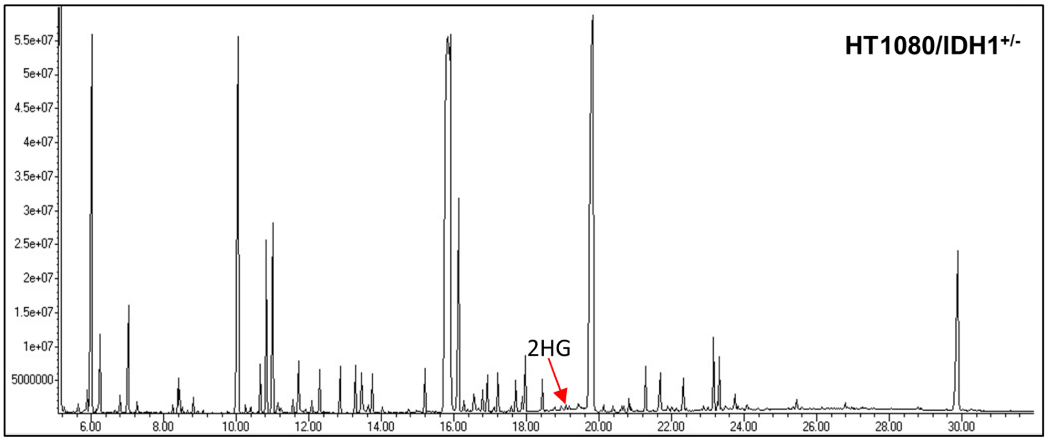


**Supplementary Figure S5: Deletion of mutant IDH1 allele eliminates D-2-HG production in HT1080 cells.** The TALEN technology was applied to generate HT1080 cells with knock-out of endogenous IDH1-R132C (HT1080/IDH1$^{+/-}$), and the intracellular 2-HG level in these cells was determined by GC-MS analysis.

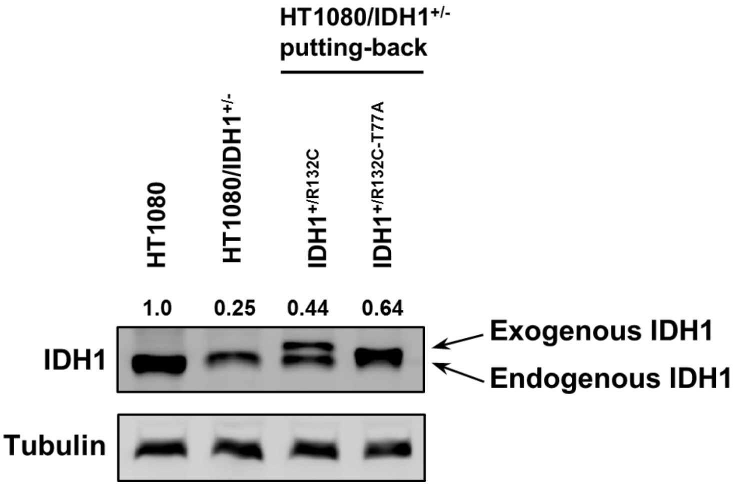


**Supplementary Figure S6: Putting-back IDH1-R132C in HT1080/IDH1$^{+/-}$ cells.** Western blotting confirmed the reduced expression of endogenous IDH1 protein and the presence of re-expressed proteins of IDH1-R132C and IDH1-R132CC-T77A.

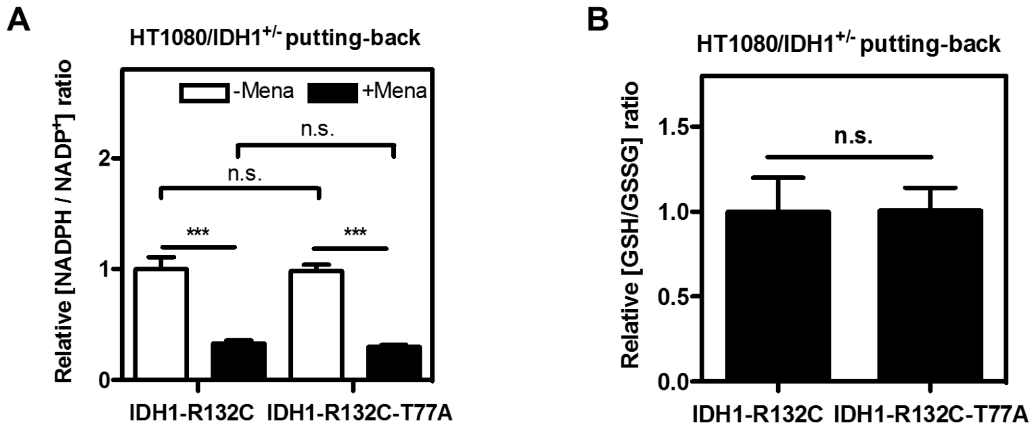


**Supplementary Figure S7: Putting-back IDH1-R132C in HT1080/IDH1$^{+/–}$ cells does not affect cellular redox status.** **(A)** IDH1-R132C mutant does not affect the ratio of NADPH/NADP+ ratio. NADPH and NADP+ were determined by the enzymatic analysis of cell extracts from HT1080/IDH1$^{+/–}$ cells putting-back IDH1-R132C or IDH1-R132C-T77A after treatment with or without menadione (25 µµM for 60 min). **(B)** IDH1-R132C mutant does not affect the ratio of GSH/GSSH. GSH and GSSG were determined by LC-MS/MS analysis in HT1080/IDH1$^{+/–}$ cells putting-back IDH1-R132C and IDH1-R132C-T77A. Shown are average values with standard error (SEM) of triplicated experiments. ***denotes $p < 0.001$ for the indicated comparison. n.s.=not significant.

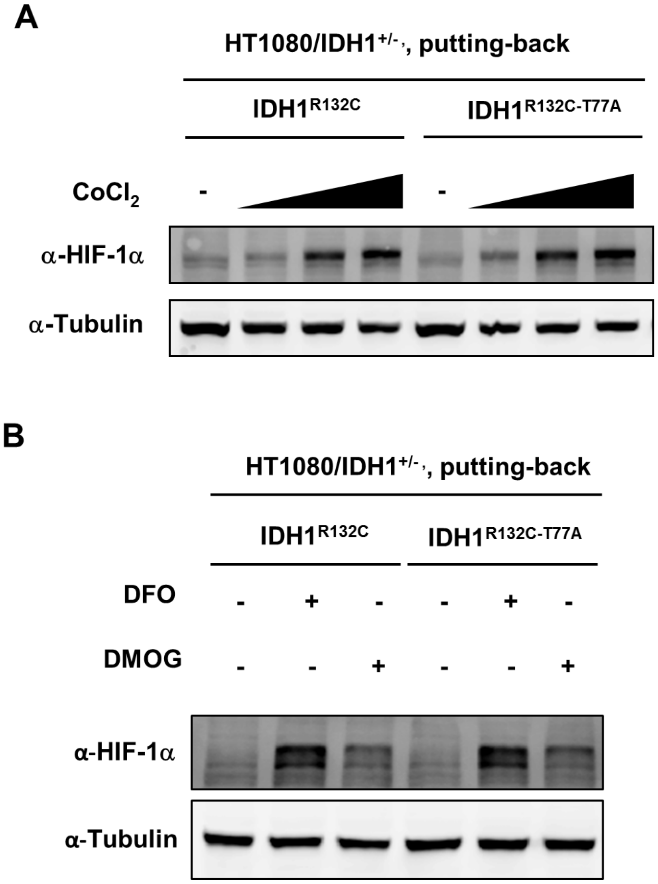


**Supplementary Figure S8: Putting-back IDH1-R132C in HT1080/IDH1$^{+/–}$ cells does not affect HIF-1α protein levels.** HT1080/IDH1$^{+/–}$ putting-back cells were treated without or with $CoCl_2$ (200 µM) **(A)**, DFO (0.5 mM) **(B)** or DMOG (1 mM) for 6 hours. The protein level of HIF-1ααwas determined by western blotting. Tubulin was detected as a loading control.

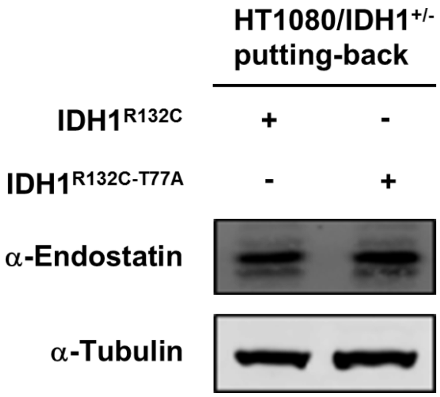


**Supplementary Figure S9: Putting-back IDH1-R132C in HT1080/IDH1$^{+/-}$ cells does not affect Endostatin protein levels.** The protein level of Endostatin in HT1080/IDH1$^{+/-}$ putting-back cells was detected by western blotting. Tubulin was detected as a loading control.

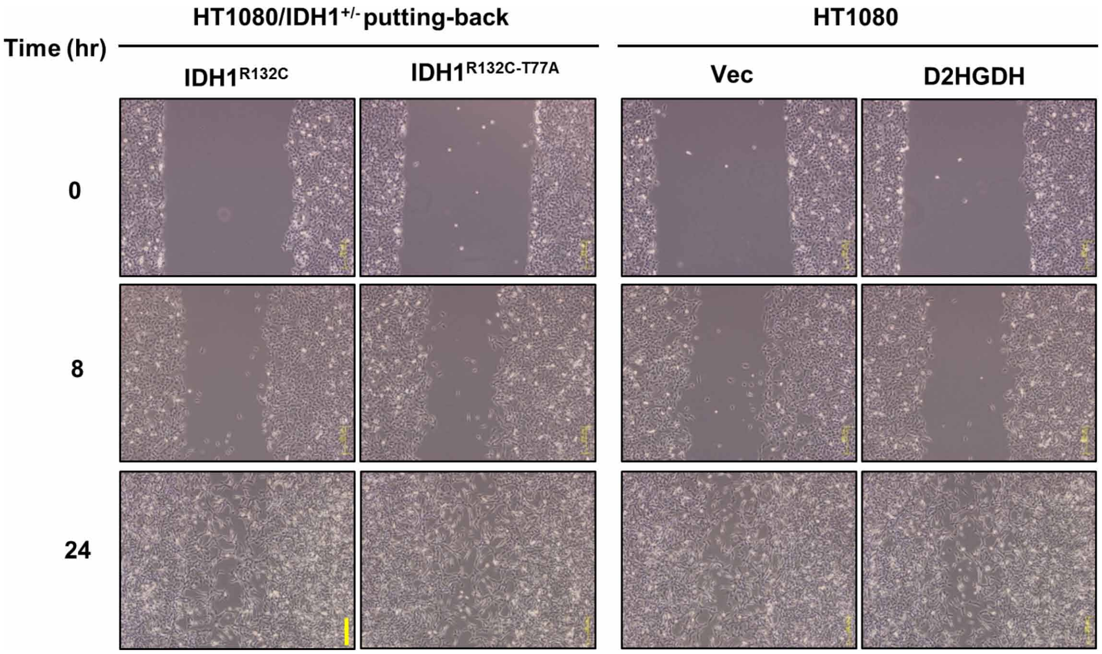


**Supplementary Figure S10: D-2-HG does not affect cell migration in HT1080 cells.** *In vitro* scratch assay was performed in HT1080/IDH1$^{+/-}$ putting-back cells, and cell motility during wound healing was carefully monitored at the indicated time points. Representative pictures were shown. Scale bar is 200 μm.
